# Health literacy in healthy adults: A systematic review of recent evidence

**DOI:** 10.1016/j.aprim.2025.103300

**Published:** 2025-07-17

**Authors:** M. Lourdes Gonçalves-Fernández, Margarita Pino-Juste

**Affiliations:** Currently Pursuing the 4th Year of a PhD Program at the University of Vigo, Spain

**Keywords:** Health literacy, Public health, Healthy adults, Systematic review, Health disparities, HLQ, Alfabetización en salud, Salud pública, Adultos sanos, Revisión sistemática, Desigualdades en salud, HLQ

## Abstract

**Objective:**

To evaluate the evidence published in the last five years on health literacy (HL) in healthy adults, analyzing reported levels, methodologies, associated factors, and publication trends across journals and editors.

**Design:**

A systematic review following PRISMA guidelines.

**Data sources:**

Electronic databases PubMed and Web of Science were searched using MeSH terms.

**Selection of studies:**

Studies published between 2018 and 2024 focusing on HL in healthy adults were included. Exclusion criteria comprised studies on populations with specific pathologies, minors, or studies conducted in restricted settings.

**Data extraction:**

Key variables extracted included sample size, age, measurement tools, study design, HL levels, and associated sociodemographic factors.

**Results:**

A total of 45 articles were included. The most frequently used instruments were the HLQ and HLS-EU-Q, although standardization was lacking. HL levels varied widely and were influenced by education, gender, and age. Most studies were cross-sectional, limiting causal interpretation. 56% of studies were from high-income countries, revealing geographical imbalance. No consistent editorial or journal focus on HL in healthy adults was observed.

**Conclusions:**

There has been substantial progress in the study of HL among healthy adults. However, methodological heterogeneity and geographical limitations restrict the generalizability of results. Future research should prioritize standardization of tools, longitudinal designs, and inclusion of low- and middle-income countries to address global HL disparities.

## Abstract (es)

**Objetivo:**

Evaluar la evidencia publicada en los últimos cinco años sobre la alfabetización en salud (AS) en adultos sanos, analizando los niveles reportados, metodologías, factores asociados y tendencias de publicación en revistas y editoriales.

**Diseño:**

Revisión sistemática siguiendo las directrices PRISMA.

**Fuentes de datos:**

Se realizaron búsquedas en las bases de datos electrónicas PubMed y Web of Science utilizando términos MeSH.

**Selección de estudios:**

Se incluyeron estudios publicados entre 2018 y 2024 centrados en la AS en adultos sanos. Se excluyeron aquellos enfocados en poblaciones con patologías específicas, menores de edad o realizados en entornos restringidos.

**Extracción de datos:**

Se extrajeron variables clave como el tamaño de la muestra, edad, instrumentos de medición, diseño del estudio, niveles de AS y factores sociodemográficos asociados.

**Resultados:**

Se incluyeron un total de 45 artículos. Los instrumentos más utilizados fueron el HLQ y el HLS-EU-Q, aunque se evidenció una falta de estandarización. Los niveles de AS variaron ampliamente y estuvieron influenciados por la educación, el género y la edad. La mayoría de los estudios fueron de corte transversal, lo que limita la interpretación causal. El 56% de los estudios procedían de países de altos ingresos, revelando un desequilibrio geográfico. No se observó un enfoque editorial o de revistas consistente sobre la AS en adultos sanos.

**Conclusiones:**

Ha habido un avance sustancial en el estudio de la AS en adultos sanos. Sin embargo, la heterogeneidad metodológica y las limitaciones geográficas restringen la generalización de los resultados. Las investigaciones futuras deben priorizar la estandarización de herramientas, diseños longitudinales e inclusión de países de ingresos bajos y medios para abordar las disparidades globales en AS.

## Introduction

Health literacy (HL) is a fundamental concept in public health, recognized for its role in empowering individuals to make informed health-related decisions and improve health outcomes.^1^ Defined as the ability to access, understand, evaluate, and apply health-related information, HL enables individuals to effectively manage their health needs in increasingly complex healthcare environments.^2^, ^3^

While extensive research has been conducted on HL in populations with chronic diseases, its implications for healthy adults remain unexplored. For this review, “healthy adults” are defined as individuals aged 18 or older without known chronic or acute health conditions requiring ongoing medical care. This population represents a critical demographic for prevention efforts, as their levels of HL influence the adoption of healthy behaviors and the effective use of healthcare services throughout their lives.

Historically, HL research has focused on clinical settings and populations with specific health needs. However, the growing interest in HL as a public health strategy has highlighted the importance of evaluating it in broader and healthier populations. In 2009, the European Health Literacy Survey (HLS-EU) developed tools to assess HL in various contexts, emphasizing the need for standardized and culturally adaptable measurement instruments.^4^, ^3^

The relevance of HL in public health extends beyond individual health outcomes. Inadequate HL is associated with significant economic burdens; for example, in the United States, it contributes to an estimated $73 billion in annual healthcare costs due to inefficiencies in resource utilization (Vernon et al., 2007). Additionally, disparities in HL disproportionately affect vulnerable groups, including those with lower educational levels or socioeconomic status, further exacerbating health inequalities.^5^, ^6^

This systematic review addresses the gap in research on HL in healthy adults by synthesizing evidence from the past five years. Its objective is to evaluate HL levels, methodologies, and associated factors to provide a comprehensive understanding of its role in public health and inform future interventions targeted at this critical population.

## Justification

Health literacy is a key determinant of public health, as it directly influences individuals’ ability to access, understand, and use health-related information, enabling them to make informed decisions and maintain their well-being. Investigating HL in healthy adults is crucial for the following reasons: Insufficient HL is associated with health outcomes, including a higher incidence of chronic diseases.^7^, ^2^ Disparities in HL are closely related to health inequalities, disproportionately affecting vulnerable groups.^8^, ^9^ Lack of HL can result in inefficient resource use, such as unnecessary hospitalizations and repeated visits.^10^

Health literacy is not only essential for individual health but also has a significant impact on the efficiency of health systems and the reduction of social inequalities. Studying it in healthy adults contributes to the development of inclusive and sustainable policies that benefit society.

## Objective

The main objective of this systematic review is to analyze studies published in the last five years on health literacy (HL) in healthy adults. It aims to evaluate reported levels of HL, the methodologies used, and the study designs applied to examine this concept.

Additionally, this work seeks to provide consolidated evidence that serves as a foundation to:

∘ Formulate effective health policies.
∘ Design educational programs aimed at improving HL.
∘ Develop innovative communication strategies.

Ultimately, the study aspires to contribute to the strengthening of public health and community well-being by enhancing HL.

## Methodology

### Study design

This systematic review was developed to synthesize and critically evaluate the published evidence on health literacy (HL) among healthy adults. In adherence to the PRISMA (Preferred Reporting Items for Systematic Reviews and Meta-Analyses) guidelines, the methodology ensured rigor, transparency, and reproducibility throughout each stage of the review process.

### Protocol and registration

Although this review followed PRISMA guidelines, it was not registered in PROSPERO due to the absence of a specific focus on chronic disease populations or clinical interventions—criteria prioritized by PROSPERO. Future reviews may benefit from formal registration to further enhance methodological transparency and reduce research duplication.

### Eligibility criteria

The research question was structured using the PICO framework:

• P (Population): Adults aged 18 years or older, defined as “healthy adults” without known chronic or acute conditions requiring ongoing medical care.
• I (Intervention): Measurement and evaluation of health literacy levels.
• C (Comparison): Not applicable.
• O (Outcomes): Reported health literacy levels and methodologies employed.

Inclusion criteria:

• Studies published between 2018 and 2024.
• Studies focusing on health literacy in adult populations.
• Use of validated instruments to measure HL.

Exclusion criteria:

• Studies involving minors.
• Studies exclusively targeting populations with specific pathologies (e.g., pregnant women, hospitalized patients).
• Theoretical or non-empirical studies.

### Information sources and search strategy

The literature search was conducted in two primary databases: PubMed and Web of Science, accessed via the University of Vigo library system. The strategy employed Medical Subject Headings (MeSH) to refine and increase the precision of results. Boolean operators (AND, OR) were used to combine the terms: “*Health Literacy,” “Healthy Adults,”* and “*Measurement Tools.”*

Terms not aligned with the study objectives—such as “*parents,” “patients,”* and “*chronic diseases”*—were excluded. To ensure consistency, the search was limited to studies published within the past five years (2018–2024). Any initial discrepancy regarding the time frame was corrected during the selection process.

### Study selection

Articles were independently reviewed at three levels—title, abstract, and full-text—by the authors. Any discrepancies in selection were resolved through discussion and consensus. Reference management and study tracking were facilitated using Zotero software.

### Data extraction

Essential data were extracted from each selected study and compiled into summary tables including the following elements:

∘ Author(s), publication year, and country of origin
∘ Sample size and age range of participants
∘ Health literacy measurement instruments
∘ Study design and principal findings

### Risk of bias assessment

Risk of bias was evaluated using standardized tools appropriate to each study design. For cross-sectional studies, sources of potential bias such as selection methods and measurement variability were critically assessed. Disagreements among reviewers were resolved through deliberation and consensus.

### Synthesis and analysis

The extracted data were synthesized through descriptive tables and narrative summaries. This dual approach enabled the identification of trends, inconsistencies, and methodological gaps across studies, offering a comprehensive understanding of the current evidence on HL in healthy adult populations.

## Results

### Study selection

The initial search across databases yielded 371 articles. After the removal of 23 duplicates, 348 articles remained for title and abstract screening. Of these, 277 were excluded for failing to meet the inclusion criteria—most commonly due to a focus on specific pathologies or theoretical frameworks unrelated to the objectives of this review.

Following a full-text assessment of 71 articles, 26 were excluded due to methodological or relevance concerns. Ultimately, 45 studies met all criteria and were included in the final synthesis.

**Figure 1:**
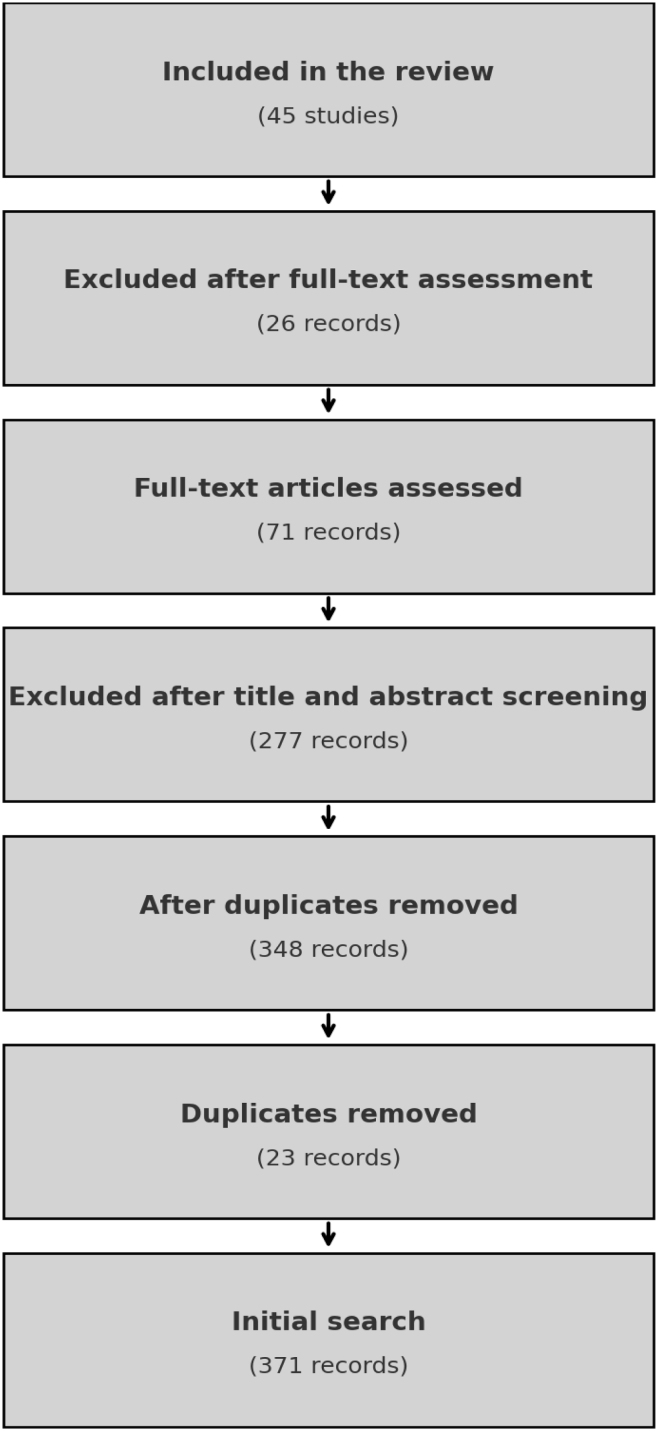
PRISMA flow diagram.

The updated PRISMA flow diagram (Fig. 1), developed in accordance with the PRISMA 2020 guidelines, visually represents the article selection process. See Fig. 1: PRISMA Flow Diagram.

### Characteristics of included studies

The studies included in this review span diverse geographical regions, study designs, and population groups. See Table 1. Notably, 56% were conducted in high-income countries, while only a limited number focused on low- and middle-income countries (LMICs).

**Table 1:** Methodological features and main results of the selected studies.

Author/country/year of publication	Sample size	Population age	Instrument	Journal	Study method	Impact factor	Quartile	HL categories	HL results	Related variables
Iran, 2019^11^	*N* = 700	18–65	HLQ	Journal of Education and Health Promotion	Cross-sectional, Descriptive	1.60	Q3	Inadequate, Marginal, Adequate, Excellent	HL 18% inadequate, 27.7% marginal, 34.9% adequate, 14.7% excellent	Age, educational level, marital status, occupation, disease history (*p* < 0.001)
United Kingdom, 2020^12^	*N* = 2309	18–75	HLQ	BMC Public Health	Cross-sectional, Descriptive	4.135	Q2	Adequate, Marginal	19.4% had difficulty reading health information, 23.2% discussed health concerns with professionals	Information understanding (3.98), Participation ability (3.83); both scored a modal of 4
USA, 2019^13^	*N* = 142	18–65	REALM, SAHLSA	Health Lit Res Pract	Cross-sectional	4.00	Q1	Marginal, Adequate	Average REALM 63.65, SAHLSA 45.45	Education-income correlation (*r* = .44), English proficiency (*r* = .45), U.S. nationality with SAHLSA (*r* = −.46)
Iran, 2022^14^	*N* = 261	18–65	Helia	BMC Public Health	Cross-sectional, Cluster Sampling	4.135	Q1	Inadequate, Adequate	Adequate HL 81.2%, Inadequate HL 18.8%	Nutritional literacy: Adequate 37.9%, Inadequate 62.1%
Czech Republic, 2021^15^	*N* = 303	18–64	HLS-EU-16	Public Health	Cross-sectional, Quantitative Descriptive	4.984	Q2	Inadequate, Adequate	Inadequate HL 49%, Adequate HL 51%	41.6% with one or more chronic illnesses, 20.5% smoke, 37.6% report health limitations
Taiwan, 2021^16^	*N* = 1297	>20	HLS-EU-Q (Chinese version)	Asia-Pacific Journal of Public Health	Cross-sectional	2.27	Q3	Moderate, Inadequate	Average HL 2.90 (moderate)	Lower education linked to lower HL; living with children under 12 associated with lower HL
Turkey, 2021^17^	*N* = 387	18–61	AHLS, 23 Sezer's Questions	Journal of Pharmaceutical Research International	Cross-sectional, Random Sampling	0.036	Q4	Inadequate, Adequate	Inadequate HL 92.2%, Adequate HL 7.8%	Significant relationship between HL and residence, age group, educational level, and occupation (*p* < 0.001)
Australia, 2022^18^	*N* = 230	>18	HLQ	Australian Journal of Health Promotion	Cross-sectional	2.033	Q3	High, Low	High HLQ in “understanding health information” (*M* = 4.19), low in “information evaluation” (*M* = 2.97)	Non-native English speakers scored lower in 7 out of 9 HLQ domains
China, 2023^19^	*N* = 500	>65	HLS-EU-Q16	Frontiers in Public Health	Cross-sectional	3.707	Q1	Basic, High	Average HL 75.25 ± 12.33, 6.33% of adults with basic HL	Association between HL and quality of life, social support
USA, 2023^20^	*N* = 98	>18	HLS-EU-Q16	Journal of Health Literacy	Cross-sectional	2.175	Q2	Sufficient, Problematic, Inadequate	Sufficient HL 58%, Problematic 27%, Inadequate 15%	Educational level, access to health resources, age
China, 2023^21^	*N* = 350	>65	Adapted HL	BMC Geriatrics	Cross-sectional	4.214	Q1	Adequate, Limited	Limited HL 68%, Adequate HL 32%	Social support, health-related quality of life, age, and gender
Iran, 2020^22^	*N* = 600	18–65	HLQ	Journal of Health Education and Promotion	Cross-sectional	Not reported	Q3	Moderate, High	Moderate HL 70%, High HL 29%	Young women, singles, and those with government jobs had higher HL levels
Hungary, 2021^23^	*N* = 1206	>18	HLQ	European Journal of Public Health	Cross-sectional	4.424	Q2	Adequate, Limited	Adequate HL 86.8% (95% CI 85.5–88.1), 13.3% reported difficulties in health service communication	Individuals with low socioeconomic status and chronic illnesses face greater difficulties in health communication
Turkey, 2020^24^	*N* = 1672	18–87	PHLKS	European Journal of Public Health	Cross-sectional, Cronbach's Alpha = 0.72	4.424	Q2	Inadequate, Adequate	Average score 12.38 (maximum 13); correct response rate 27.8%, indicating low public health literacy	Higher educational levels were associated with higher public health literacy levels
China, 2019^25^	*N* = 2475	>18	HLQ	Southeast Asian Journal of Tropical Medicine and Public Health	Cross-sectional, Construct Validity = 0.78	2.27	Q3	Inadequate, Adequate	Inadequate HL 83.6%, Adequate HL 16.4%	60% believe adequate HL is essential for health; 70% associate poor self-management education with poor health
Greece, 2020^8^	*N* = 1281	>18	HLS-EU-47	Mediterranean Journal of Nutrition and Metabolism	Cross-sectional	Not reported	Q4	Adequate, Marginal	Average HL 32.28 for men, 22.11 for women	Age and sex predict HL levels; individuals aged 56–65 had higher HL levels (*p* = 0.023)
Taiwan, 2020^26^	*N* = 161	>65	HLS-EU-Q47	Medicine-Lithuania	Cross-sectional	2.948	Q2	Inadequate, Adequate	Inadequate HL 57.76%, Adequate HL 42.23%	57.76% had inadequate or problematic HL. Average HL index was 30.83
Korea, 2021^27^	*N* = 1521	70–84	BRFSS	International Journal of Environmental Research and Public Health	Prospective cohort	4.614	Q2	Limited, Not Limited	Limited HL 68%, Not Limited HL 32%	Limited HL increases frailty risk (RRR = 1.45, *p* = 0.02) and pre-frailty (RRR = 2.03, *p* = 0.01)
USA, 2018^28^	*N* = 2573	>50	PIAAC	Educational Gerontology	Nationally representative sample	1.389	Q3	Mediated, Non-Mediated	Literacy skills mediate 31.89% of the education-health relationship	HL and literacy activities mediate the relationship between education and health outcomes
Germany, 2021^29^	*N* = 565	18–25	Lenartz HL	International Health Promotion	Cross-sectional	3.734	Q2	Low, High	Constructs of the HL structural model ranged from 2.6 to 3.0	Association observed between the HL model and work capacity in young employees
China, 2019^30^	*N* = 992	>65	HLQ	Medicine	Cross-sectional, Descriptive Analytical	1.817	Q3	Associated, Not Associated	HL associated with productive aging (b = 0.676, 95% CI 0.604–0.748)	HL has direct associations with social support (beta = 0.327, 95% CI: 0.175–0.479)
Iran, 2020^31^	*N* = 1665	>18	TOFHLA	Salmand-Iranian Journal of Aging	Systematic Review, Meta-analysis (6 articles)	Not reported	Q4	Inadequate, Limited	Average inadequate HL in 45.8% of older adults	HL higher in men (57.24%) compared to women (44.28%)
Ghana, 2019^32^	*N* = 521	>18	FHL	International Journal of Environmental Research and Public Health	Cross-sectional, Descriptive Analytical	4.614	Q2	Sufficient, Problematic, Inadequate	Sufficient HL 37.2%, Problematic 30.1%, Inadequate 32.6%	Positive relationship between HL and health status, particularly with high informational support (*β* = 0.315, *t* = 3.067, *p* = 0.002)
Korea, 2022^33^	*N* = 2808	70–84	BHLS	Geriatrics & Gerontology International	Longitudinal, 2 years	3.387	Q3	Limited, Adequate	Limited HL 59.15%, Adequate HL 40.95%	Limited HL associated with 1.4 times greater risk of developing pre-frailty over two years
Switzerland, 2019^34^	*N* = 5959	18–25, males in military service	YASS	International Journal of Public Health	Cross-sectional, Longitudinal	5.1	Q2	Associated, Not Associated	OR showed significant associations with self-rated health, depression tendency, and physical health	HL showed significant associations in six logistic regression models (1.16 ≥ OR ≥ 1.04, *p* < 0.001)
USA, 2022^35^	*N* = 9 articles	>50	6 measurement instruments	Geriatric Nursing	Systematic Review, Meta-analysis	2.525	Q4	Associated, Not Associated	Low HL associated with vision and hearing loss	HL cannot be interpreted with a single approach due to variability in instruments
Japan, 2021^36^	*N* = 218	65–86	NVS	Nihon Ronen Igakkai Zasshi	Longitudinal	0.14	Q4	Inadequate, Adequate	Inadequate HL 17.9%, Adequate HL 82.1%	HL is a protective factor against frailty; older adults with higher HL have lower frailty risk
Sweden, 2022^37^	*N* = 1500	>77	Communicative and Critical HL Scale	European Journal of Public Health	Cross-sectional	3.367	Q1	Inadequate, Adequate	Inadequate HL 49%, Adequate HL 51%	HL in older adults varies with age, educational level, and visual and cognitive ability
China, 2021^38^	*N* = 995	>65	HLQ	Frontiers in Public Health	Cross-sectional	5.99	Q1	Adequate, Inadequate	Inadequate HL 91.5%, Adequate HL 8.5%	HL has a direct positive effect on productive aging; education and income have direct positive effects on HL
Japan, 2022^39^	*N* = 2697	>18	HLQ (14 items)	Asia-Pacific Journal of Public Health	Cross-sectional	2.270	Q3	Functional, Communicative, Critical	Total HL 49.8; Functional 19.0, Communicative 17.1, Critical 13.7	Higher HL associated with continuation of physical activity during the pandemic
USA, 2021^40^	*N* = 83	≥65	PFFS	Gerontology and Geriatric Medicine	Cross-sectional	Not reported	Q4	Inadequate, Adequate	Inadequate HL 69.9%, Adequate HL 30.1%	PFFS is valid and feasible for assessing frailty in older veterans with varying levels of HL
USA, 2021^41^	*N* = 15	>65	S-TOFHLA	American Occupational Therapy Association, Inc	Cross-sectional	2.813	Q1	Marginal, Not Limited	All participants significantly improved HL scores when time restrictions were removed	Removing time restrictions can significantly enhance HL scores
Taiwan, 2022^42^	*N* = 7702	>18	HLQ (9 items)	International Journal of Environmental Research and Public Health	Longitudinal	4.614	Q2	Inadequate, Adequate	Inadequate HL 25.3%, Adequate HL 74.7%	Deficient HL is a risk factor for frailty
Ukraine, 2020^43^	*N* = 100	>18	HLS-EU-16	European Journal of Public Health	Snowball Sampling	4.424	Q2	Low, Medium	Average HL score 11.06 in Ukraine, 11.44 in Poland	No significant differences in HL between the two groups
Australia, 2023^44^	*N* = 1578	≥65	Australian Health Literacy Survey 2018	Health Promotion Journal of Australia	Regression Analysis	2.500	Q2	Health Literacy, Disparities in Care	20% of participants scored high in health literacy. Ages 65–69: 60% with adequate HL; ≥70 years: 75% with adequate HL	Better scores associated with English proficiency and higher educational levels; chronic illnesses (cancer, hypertension, arthritis), psychological distress, low English proficiency
USA, 2023^45^	*N* = 89	Mean 53.1	NVS (Newest Vital Sign) Adapted to C-NVS (self-administration)	PEC Innovation	Randomized Clinical Trial	1.60	Q3	Inadequate, Adequate	75.6% Adequate, 24.4% Inadequate	Age, educational level, health insurance, race, ethnicity
Hong Kong, 2023^46^	*N* = 433	≥18 (Mean 50)	HLS-Hong Kong	Frontiers in Public Health	Cross-sectional Survey	3.15	Q2	Functional, Interactive, Critical	5 key HL factors explained 53% of total variance. Higher HL scores correlated with better health status	Education, self-reported health status, physical activity, monthly income, mental health
Germany, 2024^47^	*N* = 3011 (Adults)	≥16	HLS-EU-Q16	Frontiers in Psychology	Cross-sectional Study	2.6	Q1	Inadequate or Problematic, Adequate	Inadequate or problematic HL associated with higher likelihood of eating disorders. Negative body image linked to higher rates of eating disorders	Gender, age, social status, educational level, body image
Japan, 2023^48^	*N* = 6230	≥65 years	HLQ Scale	Aging Clin Exp Res	Cross-sectional	4.1	Q2	High, Medium, Low	High community HL associated with lower frailty prevalence (OR: 0.28, 95% CI). Frailty prevalence: 26.2%	Education, social networks, BMI, depressive symptoms
Brazil, 2024^49^	*N* = 35	Median 50 years	TOFHLA	Alzheimer's Dementia	Cross-sectional	3.5	Q1	Low, Medium	HL correlated with hippocampal connectivity. No compensation with memory. Proposed HL-based cognitive intervention to prevent decline	Brain structure, education level, structural racism
USA, 2023^50^	*N* = 174	≥62 years	Adapted HLQ	Cardiovascular Nursing Journal	Cross-sectional	2.8	Q2	High, Medium, Low	Resilience and HL predict medication adherence in heart failure patients	Resilience, social support, depression, race
France, Sub-Saharan Africa, 2023^51^	N/A	N/A	Systematic review of studies in low- and middle-income countries (LMICs)	International Journal of Noncommunicable Diseases	Narrative Review	2.76	Q2	General	Low HL in >50% of studies; associated with 30%-40% less health service utilization and increased morbidity and mortality	Social determinants, noncommunicable diseases (NCDs)
Portugal, 2023^52^	N/A	Adults	NUTLY: Photo-based instrument for measuring nutrition literacy	European Journal of Public Health	Instrument development and validation	4.06	Q1	Nutrition	Internal reliability coefficient (*α* = 0.82); significant correlation with nutritional education (*r* = 0.68, *p* < 0.01)	Education, visual and interpretative skills
China, 2023^53^	*N* = 426	Lactating women	NLAI-L: Instrument for measuring nutrition literacy in lactating women	Nutrients	Instrument development and validation	6.70	Q1	Knowledge, Skills, Behaviors	HL mean: 46.0 ± 9.3; *α* coefficient = 0.84; *χ*^2^/df = 2.28, RMSEA = 0.057 (acceptable validity)	Age, educational level, occupation, postnatal period
China, 2023^54^	*N* = 471	≥60 years	HL Scale for Chronic Patients; SEMCD; SF-12	Frontiers in Public Health	Cross-sectional study with moderated mediation model	6.461	Q1	Access, Understanding, Evaluation, and Application of Health Information	Positive HL associated with better physical and mental health; partial mediation by self-efficacy (26.9% of total effect)	Disease duration, self-efficacy, gender, age, occupation

The Health Literacy Questionnaire (HLQ) emerged as the most commonly used instrument, followed by the HLS-EU-Q and other validated tools. Despite their frequent use, a lack of standardization in measurement approaches was apparent.

In terms of study design:

∘ 28 studies were cross-sectional
∘ 4 were longitudinal
∘ 3 were systematic reviews with meta-analyses

### Risk of bias assessment

The assessment of risk of bias revealed several recurring methodological concerns:

∘ Selection bias: Many studies did not employ random sampling, thereby limiting representativeness.
∘ Measurement bias: Variability in HL tools and their cultural adaptations posed challenges for comparability.
∘ Reporting bias: Only 60% of studies explicitly acknowledged methodological limitations.

### Key findings

#### Levels of health literacy

HL levels varied significantly across studies:

• Adequate HL: Reported in 50% to 80% of participants in high-income settings.
• Inadequate HL: More commonly reported in LMICs, with some studies noting rates as high as 40%.

#### Influential factors

Several sociodemographic variables were consistently associated with HL levels:

∘ Educational attainment: Higher education levels correlated positively with HL across all studies.
∘ Gender: Women generally exhibited higher HL levels than men.
∘ Age: Both younger^17^, ^18^, ^19^, ^20^, ^21^, ^22^, ^23^, ^24^, ^25^, ^26^, ^27^, ^28^, ^29^, ^30^, ^31^, ^32^, ^33^, ^34^ and older adults (65+) tended to have lower HL than middle-aged adults.
∘ Language: Participants assessed in their native language showed better HL outcomes, emphasizing the need for culturally adapted tools.

#### Methodological trends

∘ Population definition: Most studies did not explicitly distinguish “healthy adults” from the general population, revealing a gap in targeted research.
∘ Instrument standardization: While tools such as the HLQ and HLS-EU-Q are widely used, no universally accepted gold standard exists.

### Meta-analysis of inadequate health literacy

A meta-analysis was conducted on 16 studies that reported quantifiable prevalence rates of inadequate health literacy among healthy adults.

**Figure 2:**
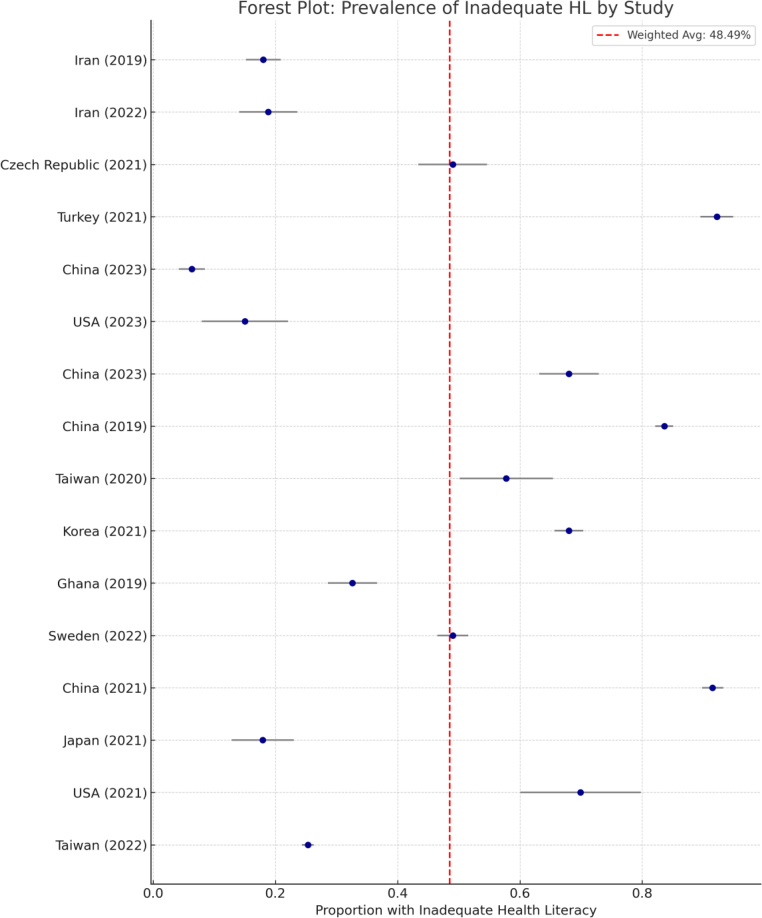
Forest plot showing prevalence of inadequate health literacy across included studies (*n* = 16). The red line indicates the pooled prevalence (48.5%).

The pooled prevalence, calculated using weighted averages based on sample sizes, was 48.5%, indicating that nearly half of the participants across these studies demonstrated inadequate health literacy (see Fig. 2).

Fig. 2 presents a forest plot displaying the prevalence of inadequate health literacy in each of the 16 included studies. The red line represents the overall pooled prevalence of 48.5%.

**Figure 3:**
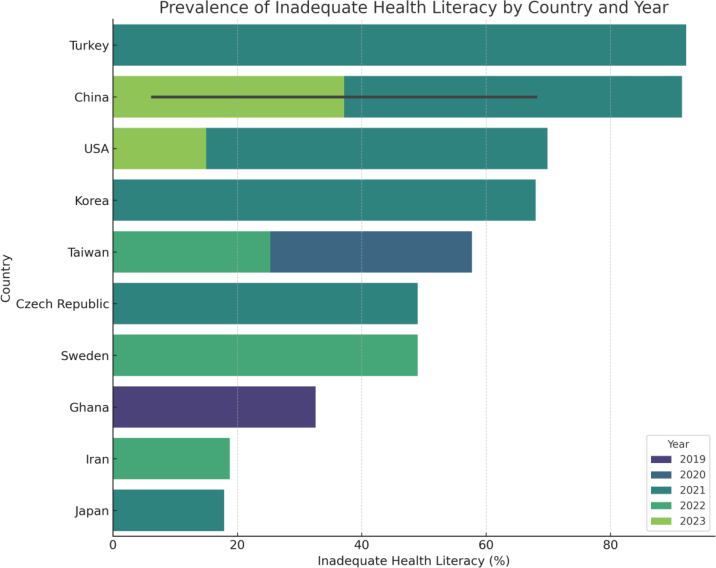
Bar plot of inadequate health literacy prevalence by country and year of study publication.

See Fig. 3, which complements this analysis with a bar chart illustrating the prevalence by country and year of publication, highlighting key geographical and temporal variations.

Prevalence rates varied substantially across studies:

• Lowest: 6.3% (China, 2023)
• Highest: 92.2% (Turkey, 2021)

This variability underscores the influence of contextual factors such as geographic region, sociodemographic composition, and the measurement instruments used.

## Discussion

Health literacy (HL) has emerged as a fundamental determinant of public health, influencing health outcomes, healthcare utilization, and health equity.^55^, ^2^ This systematic review synthesizes findings on HL in healthy adults, providing critical insights into methodologies, results, and gaps in existing research.

A key finding of this review is the growing emphasis on measuring HL using validated tools such as the HLQ and HLS-EU-Q.^4^, ^3^ These instruments have facilitated a deeper understanding of HL across diverse populations, but the lack of a universally accepted tool continues to hinder comparability between studies. Beyond tool standardization, notable advancements include: Consistent correlations between health literacy and educational attainment, gender, and socioeconomic status highlight the role of structural inequalities in shaping HL levels.^9^, ^8^ The predominance of studies from high-income countries underscores the need to expand HL research to low- and middle-income regions, where disparities are likely more pronounced.^51^ Several studies emphasized the importance of culturally adapted HL tools, especially in linguistically diverse populations.^18^, ^13^

This review confirms gender-based differences in health literacy, with women consistently demonstrating higher HL levels than men. This may reflect differences in health-seeking behaviors, social roles, and educational opportunities. For instance, studies in Europe and Asia reported higher rates of health information engagement among women.^8^, ^17^ These findings suggest that interventions targeting men could address critical gaps in HL.

Unlike populations with chronic conditions, where HL is often studied in the context of disease management,^7^ HL in healthy adults focuses on prevention and health promotion. This distinction is significant, as healthy adults may lack the immediate motivation to engage with health information, highlighting the need for tailored strategies to enhance HL in this group. Additionally, studies on individuals with chronic conditions often report stronger associations between HL and clinical outcomes, suggesting that HL interventions in healthy adults should prioritize long-term benefits and preventive care.^48^, ^30^

The predominance of cross-sectional designs limits the ability to establish causal relationships, underscoring the need for longitudinal studies.^33^, ^27^ These studies could explore how health literacy evolves over the lifespan and its impact on health behaviors and outcomes. Furthermore, few studies explicitly addressed the role of digital health literacy, an area of growing importance in an increasingly digitalized healthcare environment.^35^

The findings of this review have significant implications for health policy and practice: Addressing HL disparities requires targeted interventions for populations with lower educational levels and those in low-income settings.^7^, ^2^ Health promotion campaigns should consider gender-specific approaches to improve engagement among men.^8^, ^56^ Developing multidimensional and culturally adaptable HL assessment tools is essential for robust data collection and comparability.^4^, ^3^

While this review provides a comprehensive synthesis, it is not without limitations. The focus on healthy adults may limit the generalizability to other populations. Additionally, the lack of studies from low- and middle-income countries restricts the global applicability of the findings. Future research should prioritize these regions to ensure a more equitable representation of HL worldwide.^51^

The meta-analysis revealed that nearly half of the healthy adult population in the reviewed studies exhibited inadequate HL. This finding underscores the urgency of implementing targeted HL interventions at a population level, especially in countries with the highest reported rates.

The substantial heterogeneity across studies may be attributed to varying measurement instruments, inconsistent definitions of HL, and sociocultural differences. These results align with previous evidence linking HL with education, age, and region. Importantly, the results reinforce the need for standardized, culturally adapted HL tools to ensure global comparability and actionable insights.

## Conclusions

This systematic review provides a comprehensive synthesis of recent research on health literacy (HL) in healthy adults, highlighting its role as a critical determinant of public health. The findings confirm that HL is a multifaceted concept influenced by sociodemographic factors, methodological approaches, and geographical contexts.

Significant progress has been made in understanding HL in healthy adults, including: The influence of education, gender, and socioeconomic status on HL levels has been identified. These findings underscore the importance of addressing structural inequalities to promote health equity. There has been a growing use of validated HL instruments, such as the HLQ and HLS-EU-Q, which have facilitated more accurate assessments. However, the lack of standardization in measurement tools remains a challenge for comparisons between studies. There is an increasing recognition of the importance of culturally adapted HL tools and methodologies, particularly in linguistically diverse populations.

The results of this review highlight critical areas for intervention and policy development: Strategies to improve HL should prioritize vulnerable groups, including those with lower educational attainment and individuals in low-income settings. Addressing the lower HL levels observed in men requires personalized health promotion campaigns that effectively engage this population. Developing multidimensional and culturally adaptable HL assessment tools is essential for enhancing the comparability of results and supporting evidence-based policy formulation.

Future studies should address several gaps identified in this review: Understanding how HL evolves over time and its long-term impact on health behaviors requires longitudinal study designs. Expanding HL research to low- and middle-income countries is essential to provide a more comprehensive understanding of global HL trends. As healthcare systems increasingly rely on digital tools, studying the role of digital HL in healthy adults will be crucial.

This review is subject to limitations, including the predominance of cross-sectional studies, the focus on healthy adults, and a geographical bias toward high-income countries. Addressing these limitations in future research will strengthen the evidence base and support the development of effective HL interventions. In conclusion, promoting HL in healthy adults is essential for reducing health disparities, optimizing healthcare resources, and enhancing well-being. The findings of this review provide a foundation for designing specific strategies and policies that address the multifaceted nature of HL and its critical role in public health.

## Ethical approval and consent to participate

This study is a systematic review based on previously published data that is publicly accessible. Therefore, ethical approval and participant consent were not required.

## Funding

This study did not receive specific funding from public agencies, commercial sectors, or non-profit organizations.

## Conflict of interest

On behalf of all authors, the corresponding author declares that there is no conflict of interest related to this study.

## Availability of data and materials

The data used and analyzed during the present study are available in the articles cited and referenced within this manuscript.
